# Characterization of the Volatile Compounds of Zhenba Bacon at Different Process Stages Using GC–MS and GC–IMS

**DOI:** 10.3390/foods10112869

**Published:** 2021-11-19

**Authors:** Linjie Xi, Jing Zhang, Ruixiao Wu, Tian Wang, Wu Ding

**Affiliations:** 1 College of Food Science and Engineering, Northwest A&F University, Xianyang 712100, China; 15829031947@163.com (L.X.); Ruixiao_Wu@163.com (R.W.); sxsxys1997@163.com (T.W.); 2 College of Horticulture, Northwest A&F University, Xianyang 712100, China; yyzhj@nwsuaf.edu.cn

**Keywords:** Chinese bacon, volatile organic compounds, GC-MS, GC-IMS

## Abstract

Zhenba bacon (ZB), a type of Chinese traditional bacon with a long history, has attracted considerable attention in the Southwest of China for its unique flavor. To elucidate the changing course of aroma components during the process of ZB, four stages of process stages were assessed by GC–MS and GC–IMS coupled with multivariate data analysis. A total of 44 volatile compounds were identified by GC–IMS, including 5 esters, 8 alcohols, 12 aldehydes, 3 ketones, 1 furan and 2 sulfides; 40 volatile compounds were identified by GC–MS, 4 ketones, 7 phenols, 8 alcohols, 6 esters, 6 aldehydes, and 6 other compounds were detected. During the curing period, the amount and content of esters in Zhenba bacon gradually increased. Phenols appear in large quantities during the smoking period. The VOCs (volatile organic compounds) in the gallery plots were the most diverse in YZ samples, which are mainly esters. POV (peroxide value) and TBARS (thiobarbituric acid reactive substance) showed that lipid oxidation played an important role in the formation of volatile flavor components of bacon. This study provides valuable analytical data to explain the flavor formation of Zhenba bacon.

## 1. Introduction

Chinese bacon belongs to a wide range of salted, dried, and smoked meat products available in China. Improvements in people’s living standards lead to changes in their diets, and over the past few decades, smoked meat products have risen in popularity due to their unique flavor. 

Zhenba bacon is typically processed for two months, beginning in November and ending in December, based on the lunar calendar, with traditional processing methods of salting and smoking. Zhenba is located in the Qinba mountains, with temperatures at 0~10 °C in the winter, making it a suitable climate for the production of bacon. At the same time, Zhenba bacon is a geographical symbol of China with an attractive color and a unique flavor, deeply favored by consumers.

The flavor of Chinese bacon is a complex process involving a series of chemical reactions and a variety of factors. At present, most scholars at home and abroad believe that the flavor mainly follows three pathways: lipid oxidation, degradation of the precursor, and the Maillard reaction. Lipid oxidation in the formation of flavor in meat products is mainly caused by lipid oxidation [1].

The research on lipid oxidation has examined two main areas [2,3]. The first area is, the oxidation of double bonds of unsaturated fatty acid to generate peroxides, which can be further broken down to volatile carbonyl compounds. Further, hydroxyl fatty acid is hydrolyzed to produce acids and the acids undergo dehydration and cyclization to yield the final lactone compounds with meat aromas.

A second aspect is that the degradation of precursor substances mainly includes the degradation of protein and oil [4]. Of course, the Maillard reaction is one of the most important reactions resulting from the heating process [5]. It utilizes the reaction between amino acids and reduces sugars to produce a variety of volatile flavor compounds in meat, such as pyrazine and pyridine [6].

At present, research on volatile flavor substances in smoked meat mainly uses gas chromatography–mass spectrometry (GC–MS), full two-dimensional gas chromatography–time-of-flight mass spectrometry (GC × GC–TOF MS), gas chromatography–sniffing (GC–O), electronic nose technology, etc. [7,8,9,10,11]. The gas chromatography–mass spectrometry (GC–IMS) ion migration technique has been emerging in recent years as a rapid detection of volatile flavor of advanced technology, GC–IMS can obtain the flavor substances and the composition of the discriminant information and sample quality, with high sensitivity, fast detection speed, easy operation and a sample analysis at low temperature, to better reflect the existing flavor state [12] of the sample. The GC–IMS approach was implemented to detect the flavor characteristics of food volatile compounds, which are usually employed to distinguish the odor characteristics of different samples. At present, the technique is commonly applied in meat [13,14], fruits [15,16], and wine [17]. Most of the previous studies mainly focused on the aroma and quality control and grading from food. GC–IMS has been widely used in food flavor analysis [18]. However, it is seldom used in Chinese bacon, which provides a new efficient method for future research on Chinese bacon, and can be combined with other detection techniques to obtain more suitable results.

In order to provide some theoretical reference for the deep processing of Zhenba bacon and the development of food dishes, the evolution of volatile flavor substances of Zhenba bacon in different processing periods was studied by utilizing GC–IMS technology combined with GC–MS and heatmap analysis.

## 2. Materials and Methods

### 2.1. Sample Preparation

For this study, fresh pork was purchased from a market in Zhenba, Hanzhong City, Shaanxi Province, China, and then samples were sent to the Mengerjie organic pork bacon factory (Zhenba) for processing.

Bacon was processed according to the traditional procedures of Zhenba bacon production. First, a volume of 1000 mL of baijiu (Chinese liquor) was uniformly spread on the surface of the pork, and then it was cured for 3 days with 4% dry salt (g/100 g fresh sample) at 15–20 °C. After marination, all the samples were washed to remove the excess salt on the surface and then drained for a day. Finally, the samples were intermittently cold-smoked using Cyclobalanopsis glauca wood for 15 days until the meat was dried. The relative humidity during the processing depended on the climatic conditions. The different stages of Zhenba bacon (1000 g, *n* = 5 each) were vacuum packed and kept frozen at −80 °C until analyzed. All of the chemical reagents used in this study were of HPLC grade.

### 2.2. Lipid Oxidation Analysis

Lipid oxidation was assessed by measuring TBARS value and POV. TBARS were measured according to Jin et al. [19] with slight modifications. The POV of the lipid sample was determined following the method of Simsec and Kilic [20].

### 2.3. GC-MS Analysis

A head space solid-phase microextraction (HS-SPME) fiber assembly combined with a GC-MS system (GC-MS 2010 SE, SHIMADZU, Tokyo, Japan) was used for detecting volatile compounds from the selected samples. Briefly, bacon samples (3 g) were added into a headspace bottle for balance at 45 °C for 30 min, and an SPME fiber (50/30 μm CAR/PDMS/DVB extraction head, Supelco, PA, USA) was used to adsorb the volatile compounds from the vial headspace at 60 °C for 30 min. After sampling the fiber was transferred to a gas chromatography inlet and desorbed at 250 °C for 3 min. Compounds were separated in a DB-WAX capillary column (30 m × 0.25 mm × 0.25 μm, Agilent J&W, Santa Clara, CA, USA). Helium (99.999%) was used as carrier gas at a flow rate of 1 mL/min and the injection volume was 1 μL. The temperature in the GC oven was maintained at 40 °C for 3 min, and then raised to 90 °C at a rate of 5 °C min^−1^ and to 230 °C at a rate of 10 °C min^−1^. The ion source was EI and the temperature was set at 200 °C. The interface temperature was set at 250 °C. A positive ionization potential of MS was at 70 eV, detection voltage of 1000 V and emission current of 100 μA. Semi-quantitative determination was obtained by using 2-methyl-3-heptanone as an internal standard. The volatile compounds acquired were identified for the reverse match factor (similarity > 700) and retention index (RI). The C7~C30 n-alkanes were employed to calculate linear RI of volatile compounds. Five replicates were applied for each product.

### 2.4. GC-IMS Analysis

Bacon samples (2 g) were placed into a 20 mL headspace (HS) vial and closed with a magnetic cap before analyzing. Subsequently, samples were incubated at 80 °C for 20 min. After incubation, a constant head-space (200 μL) was injected into the injector automatically by a heated syringe (85 °C). Then the samples were transferred into an MXT-5 (15 m × 0.53 mm) capillary column by nitrogen (99.99%) at a programmed flow as follows: initially 2.0 mL/min, 10 mL for 10 min, 100 mL for 10 min and eventually 150 mL for 10 min. The ions of analytes ionized were directed to the drift tube with a constant temperature of 45 °C and the drift gas (nitrogen gas, 99.99% purity) was set at 150 mL/min. The final results were the averages of three replicates.

### 2.5. Statistical Analysis

IMS data were analyzed by the instrumental analysis software including LAV (from G.A.S., Dortmund, Germany version 2.0.0), Reporter, Gallery Plot as well as GC × IMS Library Search, which can be used for sample analysis from different angles. The identified compounds were characterized combing retention index (RI) and drift time with NIST Library and IMS database retrieval software from G.A.S. The analysis software of the instrument includes VOCAL and three plugins, which can analyze samples from different angles.

## 3. Results and Discussion

### 3.1. Lipid Oxidation

**Figure 1:**
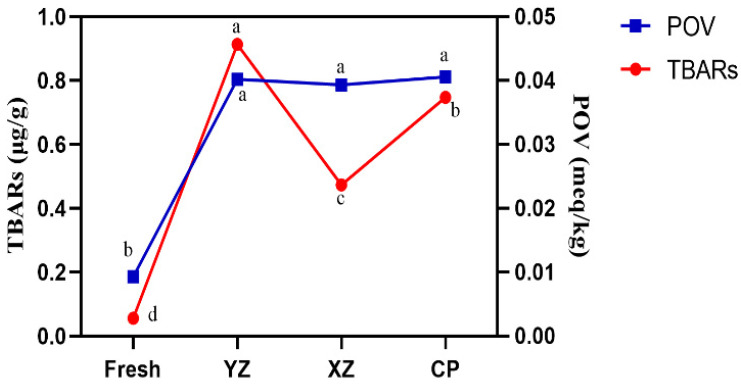
Changes of POV and TBARS content in different stages. Fresh: raw sample; YZ: cured sample; XZ: smoking sample; CP: finished sample; POV: peroxide value; TBARS: thiobarbituric acid reactive substance.

Volatile compounds are mainly composed of lipid oxidation products. POV is the measure of hydroperoxides formed during autoxidation. POV represents the degree of primary oxidation of lipids. Hydroperoxides are not stable and decompose spontaneously to form other compounds, such as aldehydes, ketones, alcohols, acids [21]. The extent of lipid peroxidation is measured by the formation of TBARS. TBARS assay detects lipid peroxidation products. The TBARS’ value refers to the reaction result of TBA with derivatives, such as malondialdehyde produced by oxidative decomposition of unsaturated fatty acids in animal fats. A high or low value indicates that the amount of secondary oxidation products, or final products, of fat has a strong correlation with the data of sensory analysis is one of the most widely used indexes to evaluate the degree of fat oxidation. Changes in POV and TBARS values at different processing stages of Zhenba bacon are shown in Figure 1. The treatments of control represent the four stages of: raw meat stage (fresh), curing stage (YZ), smoking stage (XZ), and mature stage (CP). Compared to the control (Fresh), the TBARS values of other groups significantly increased with processing time (*p* < 0.05), the TBARS value changes in bacon are due to the changes in lipid during the heating and smoking process. In general, TBARS’ values increased with increasing process period, due to the promotion of lipid oxidation and dehydration. It indicates that the degree of secondary oxidation of lipids is gradually deepening. It can be seen from the figure that after the sample is pickled, the activity of lipoxidase is enhanced due to the increase of salt, thus the secondary oxidation of lipids is intensified. For the smoking stage, as TBARS values decreased slightly, the further oxidation of aldehydes to organic alcohols or carboxylic acids is inferred.

The trends in the POV are consistent with those of the TBARS change. During bacon processing, these values were increased. The POV value reflects the content of primary lipid oxidation products, indicating that the accumulation of primary oxidation products is basically the same during processing. Jin et al. [22] found that the TBARS are mainly aldehydes, which are then degraded into volatile compounds. POV and TBARS showed that under fermentation and smoking conditions, hydroperoxide and free fatty acids were produced from the lipids of Zhenba bacon, and the lipid hydrolysates played an important role in the formation of volatile flavor components of bacon.

### 3.2. GC–IMS Topographic Plots

**Figure 2:**
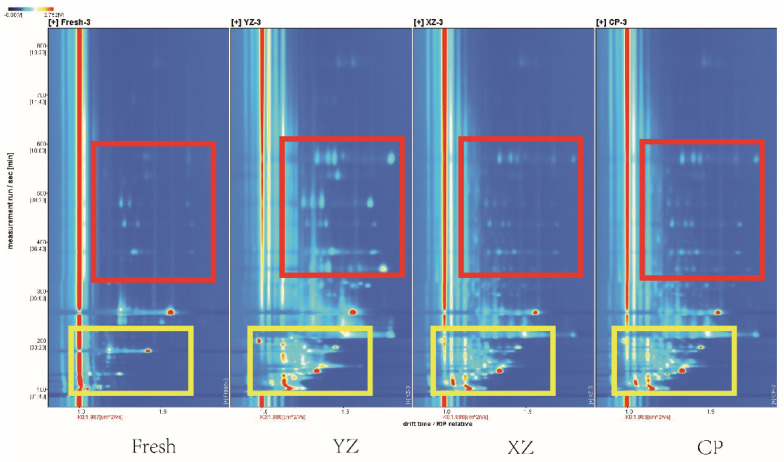
The gallery plots of VOCs from different stages using CC–IMS. Fresh: raw sample; YZ: cured sample; XZ: smoking sample; CP: finished sample; VOCs: volatile organic compounds.

The topographic plot map of the four stages of Zhenba bacon in GC–IMS is shown in Figure 2. The horizontal axis represents the migration time and the vertical axis represents each point on both sides of the reactive ion on peak (RIP peak) during retention of gas phase color spectrum representing a volatile organic matter. Colors represent the concentration of compounds; the white represents the low concentration and the red represents the high concentration. Meanwhile, deeper color indicates increased concentration. Most of the signals appeared in the retention time range from 100–400 s and drift time from 1.0–1.5 s. It can be seen from this plot that VOCs of samples at different stages are well separated from each other. The amounts of VOCs detected in the yellow box area of the fresh sample had the least content from Figure 2. The type and content of VOCs always increase with processing time. The VOCs in the red box area were the most diverse in YZ samples, while the differences were significant in other stage samples. From the GC–MS data, it is also evident that accumulation of the major constituents in YZ sample are esters. Figure 2 shows that the differences in VOCs of Zhenba bacon in different processing periods are mainly manifested in the position, number, intensity, and time of ion peaks. Compared with CP sample, the XZ sample is relatively close on the topographic map. Smoking led to the unique flavor, such as guaiacol. Combined with the data of GC–MS, it can be seen that a large number of phenolic substances are mainly produced. Meanwhile, the two stages only carried out a short burning and washing process, the influence on the change of flavor substances is less. In summary, compared with these samples, the signal intensities of substances are different among the four stages. As evident from the figure, VOCs of the different processing stages display significant differences in their topographic plots. Zhu et al. [23] found that fermentation can increase the VOCs, which can be seen intuitively in the topographic plot. Similarly, the results suggested that the identified number and concentration of VOCs in the processed samples were higher than that in the fresh samples.

### 3.3. Analysis of VOCs by Fingerprint

**Figure 3:**
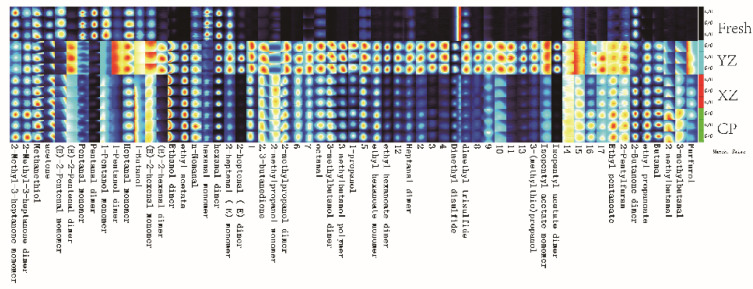
Fingerprints of VOCs isolated from different stages using GC–IMS. Each colored point represents a VOC, and different colors indicate varying concentrations, red representing higher intensity, blank meant not present. Fresh: raw sample; YZ: cured sample; XZ: smoking sample; CP: finished sample; VOCs: volatile organic compounds.

According to Figure 3, one can see that the concentration and kinds of VOCs of Zhenba bacon from four different processing stages are significantly different. It can be seen that the fresh stage contains substances that are either absent or at low concentrations in subsequent processing. The substances in region A mainly include acetone, pentanal, 1-pentanol, etc. In the flavor study, pentanal, the major straight-chain aliphatic aldehyde, contributes to the fruity and ester aromas. Due to its low flavor threshold, it could affect flavor or aroma in meat foods [24]. The variety and concentration of volatile substances were the highest in the YZ stage. The cured processing is involved in enzymatic hydrolysis of protein [25]. Therefore, it could contribute to the formation of bacon flavor. From left to right, substances in region B in the figure are mainly E-2-heptanal, 2, 3-butanedione, 2-methylpropanol, octanal, 3-methylbutanol, 1-propanol, ethyl hexanoate, heptanal, dimethyl disulphide, 3-methylthio-propanol etc. On the basis of structural considerations, it is assumed that 3-methylbutanal and 3-methylbutanol are derived from leucine while 2-methylbutanol is derived from isoleucine [26]. It is possible that these substances came from the local baijiu during the curing of bacon. 3-methylbutanol has a banana-like and pear-like aroma [27]. 2, 3-butanedione is found in numerous common flavors such as “buttery/creamy” and fruit flavors [28], and the smell is strong. It is speculated that this is the main flavor in the bacon curing period. The concentration of substances in the C region of CP sample rose to the highest, namely 2-butanone, ethyl propanoate, butanal, 2-methylbutanal and 3-methylbuanal, etc. Meanwhile, it was observed that among the four stages, XZ and CP were the most similar, while fresh and YZ had the biggest difference. Ethyl propanoate was associated with a green-sweet-fruity note [29], and butanal was the most abundant VOC in the meat samples [30].

### 3.4. Analysis of VOCs Obtained by GC–IMS and GC–MS

According to the data from the company’s built-in NIST gas phase retention index database and IMS migration time database, the volatile components were qualitatively analyzed according to the gas chromatography retention time and IMS migration time of volatile substances. A total of 44 volatile compounds were identified by GC–IMS, as detailed in Table 1, and included 5 esters, 8 alcohols, 12 aldehydes, 3 ketones, 1 furan and 2 sulfides. Thus, the volatile organic compounds in bacon are mainly aldehydes, alcohols, and esters. Due to the different concentrations of these compounds, some single compounds may produce multiple signals or spots (dimers or trimers) [31]. The retention indexes of the majority of the volatile compounds ranged from 200 to 400, which indicated that weakly polar and small-molecule volatile compounds were detected in the samples. This may be because the evaluation of advanced nondestructive testing of GC–IMS were similar to the sensory conditions of the samples [32]. Among the abovementioned compounds, 17 compounds, including acetone, 1-propanol, and 3-methylbutanal, were not detected by GC–MS. This suggested that GC–IMS technology can be used to comprehensively determine the real aroma of a sample without sample pretreatment [33]. In previous studies in Huangjiu [34], on sorghum [35], chicken [36], wheat [37], and kiwifruit [38], the differences of aroma components among samples were also revealed by GC–MS and GC–IMS.

**Figure 4:**
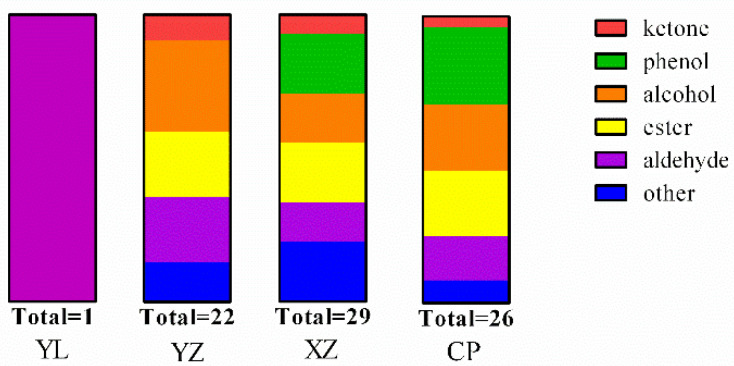
Change of VOCs species of Zhenba bacon with different stages using GC-MS. Fresh: raw sample; YZ: cured sample; XZ: smoking sample; CP: finished sample; VOCs: volatile organic compounds.

In GC–MS, 40 volatile organic compounds, 4 ketones, 7 phenols, 8 alcohols, 6 esters, 6 aldehydes, and 6 other compounds were detected (Table 2). It could be clearly seen from Figure 4 that each color represents a different type of volatile compound, and the length represents the number of species. Among them, the fresh sample only exhibits one kind of VOCs (hexanal) and XZ sample had similar flavor substance types with the CP sample. The YZ sample has more alcohol compounds and aldehyde compounds. As one of the characteristic flavor products of fat degradation, aldehydes have a low threshold value and complex taste, so their contribution to the overall flavor of Zhenba bacon is more prominent, in particular, the impact of low carbohydrate aldehydes on the flavor of bacon is more significant. Among them, saturated straight chain aldehydes generally have a strong taste of raw oil and make people vomit, which is a major characteristic flavor substance in fresh meat. In addition, hexanal can be detected in every sample. As a common flavor substance in fresh meat, hexanal has been reported many times, and it has a grass-like taste [39]. In the fresh group, only one volatile flavor substance of hexanal was detected, and in the YZ group, the maximum value was 3.496. During the curing period, a total of five aldehydes were detected, namely pentanal, hexanal, 1-nonanal, 5-methyl furfural and acetal. Some studies have found that the appropriate amount of aldehyde substances (acetal) in liquor can improve the flavor of liquor, so acetal may be used in the liquor flavor during the YZ stage. The types and contents of aldehydes are the most similar in the smoked period and finished product period, which may be caused by the covalent combination of aldehydes and proteins. With the extension of storage time, aldehydes’ flavor substances are not volatile.

Ketone was a product of lipid oxidation and hence was used as an indicator of lipid peroxidation [40]. 2,3-butanedione is a byproduct of the Maillard reaction. Because its odor threshold is low, it is likely to be an important contributor to provide buttery aroma with bacon. In the four stages, the content of 2,3-butanedione was observed only in the YZ stage. In this study, 3-Methyl-1,2-Cyclopentanedione was detected in XZ and CP stages. These methyl ketones, which are caused by autoxidation and β oxidation of fatty acids or microbial metabolism, are considered to be important contributors to the complex flavor and aroma properties of pork [41].

Most alcohols are produced by the degradation of unsaturated fats, and some may be produced by the reduction of aldehydes. In the XZ and CP samples, the concentration of 3-octenol (grass aroma and mushroom aroma) [42] and furfuryl alcohol (mildew aroma and caramel aroma) indicated a significant contribution to the bacon flavor. 3-octenol has been associated with the pathway of arachidonic acid. The content of ethanol, 1-pentanol, and 3-methyl-1-butanol has a high level in YZ, and a low level in XZ and CP samples. This could be due to the large contribution of liquor flavor substances in the YZ stage. Previous studies have shown that linear alcohols were known to originate from lipid oxidation; branched-chain alcohols were produced by a Millard-type reaction and Strecker degradation under fermentation conditions and then reduced [43].

The proportion of esters in the volatile components of Zhenba bacon was the largest, which contributed to the flavor of bacon. The formation of esters usually requires a complex reaction chain, which may come from the esterification of alcohols and carboxylic acids under the action of microorganisms [44]. Among the identified substances of esters were ethyl valerate, ethyl caproate, methyl hexanoate, ethyl butyrate, and ethyl lactate. However, content of ethyl caproate is the highest in the YZ stage, which is mainly produced in the fermentation process, and has the aroma of pineapple and banana fruits [45].

Phenolic substances are mainly generated by the decomposition of lignin in fumigating materials, which are the unique volatile flavor substances of smoked products. It is attributed mainly to the volatile compounds generated during the heating and decomposing of the smoked material, and most of the phenolic substances formed after being smoked. The findings from the present study are consistent with this idea. Phenols are only present during the XZ and CP samples, which contained 2-methylphenol, 3-methylphenol, 4-methyl guaiacol, 4-ethylguaiacol, guaiacol, and phenol. Phenolic did not differ much between these two stages.

### 3.5. Correlation between GC-MS and Lipid

**Figure 5:**
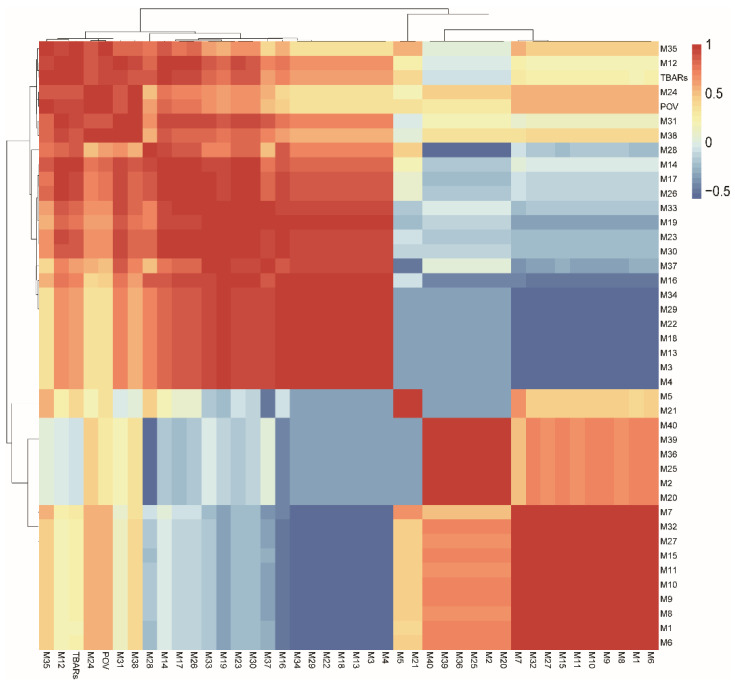
Correlations among POV, TBARS and GC–MS from different stages. The color from blue to red represents a correlation from negative to positive. Fresh: raw sample; YZ: cured sample; XZ: smoking sample; CP: finished sample; POV: peroxide value; TBARS: thiobarbituric acid reactive substance.

Lipids were one of essential flavor precursors and taste contributors that affect meat flavor and palatability. Proper lipid oxidation is a major cause for changes of flavor during storage and processing [42]. The volatile compounds are mainly produced by lipid oxidation [21]. The TBARS’ value is an index of secondary lipid oxidation, the POV value is an index of primary oxidation. Correlation analysis was used to explore the impacts of volatile flavor compounds of the Zhenba bacon during different stages and the lipid oxidation (Figure 5). A positive correlation was observed between lipid oxidation and some VOCs. Alcohols include ethanol, phenethyl alcohol, 3-octenol, 2-methyl-1-propanol, 3-methyl-1-butanol, 1-hexanol, and 1-pentanol. Aldehydes include pentanal, hexanal, 1-nonanal, 5-methyl furfural, and acetal. Esters include ethyl acetate, ethyl valerate, ethyl caproate, methyl hexanoate, and ethyl lactate.

## 4. Conclusions

This study investigated the VOCs found in the four processing stages of Zhenba bacon. A total of 40 and 44 VOCs were identified in the four samples by GC–MS and GC–IMS. The volatile organic compounds in Zhenba bacon are mainly aldehydes, alcohols, and esters. The combined use of GC–MS and GC–IMS maximizes the results by combining accurate analytical results with intuitive visualization. There were two conspicuous differences between YZ and XZ, which were the smoky attribute and lipid reaction. At the same time, esters increased in the YZ stage and phenolic compounds were derived from the XZ stage. POV and TBARS showed that under fermentation and smoking conditions, hydroperoxide and free fatty acids were produced from the lipids of Zhenba bacon, and the lipid hydrolysates played an important role in the formation of volatile flavor components of bacon. The results of this study support the view that smoking and curing are the most important stages. This research provides a necessary study of the flavor formation in Chinese bacon.

## Figures and Tables

**Table 1:** The information on detected VOCs of Zhenba bacon by GC-IMS.

Count	Compound	CAS^#^	Formula	MW ^a^	RI ^b^	Rt ^c^ [Sec]	Dt ^d^	Comment
1	Methanethiol	C74931	CH4S	48.1	504.1	96.119	1.03955	
2	Ethyl Acetate	C141786	C4H8O2	88.1	597.9	137.091	1.34375	
3	3-Methylbutanal	C590863	C5H10O	86.1	645.1	157.712	1.41113	
4	2-Methylbutanal	C96173	C5H10O	86.1	656.3	162.596	1.40419	
5	Pentanal	C110623	C5H10O	86.1	692.3	179.691	1.1862	monomer
6	Pentanal	C110623	C5H10O	86.1	693.7	180.776	1.42797	dimer
7	Ethyl Hexanoate	C123660	C8H16O2	144.2	1002.1	568.154	1.33495	monomer
8	Ethyl Hexanoate	C123660	C8H16O2	144.2	1002.5	568.932	1.80397	dimer
9	Heptanal	C111717	C7H14O	114.2	898.4	382.978	1.32916	monomer
10	Heptanal	C111717	C7H14O	114.2	896.2	379.088	1.69106	dimer
11	2-Heptenal (E)	C18829555	C7H12O	112.2	951.9	477.9	1.25967	monomer
12	2-Heptenal (E)	C18829555	C7H12O	112.2	952.7	479.456	1.67369	dimer
13	Acetone	C67641	C3H6O	58.1	530.9	107.842	1.12549	
14	1-Propanol	C71238	C3H8O	60.1	569.6	124.745	1.25108	
15	2,3-Butanedione	C431038	C4H6O2	86.1	586.2	132.008	1.17563	
16	Dimethyl Disulfide	C624920	C2H6S2	94.2	716.9	199.324	0.98008	
17	1-Pentanol	C71410	C5H12O	88.1	766.6	238.992	1.24972	monomer
18	1-Pentanol	C71410	C5H12O	88.1	766.1	238.594	1.51116	dimer
19	Furfurol	C98011	C5H4O2	96.1	820.9	293.414	1.34139	
20	(E)-2-Hexenal	C6728263	C6H10O	98.1	844	318.919	1.18372	monomer
21	(E)-2-Hexenal	C6728263	C6H10O	98.1	842.7	317.4	1.5273	dimer
22	2-Pentylfuran	C3777693	C9H14O	138.2	990.1	545.855	1.24933	
23	Ethanol	C64175	C2H6O	46.1	520.2	103.167	1.04743	monomer
24	Ethanol	C64175	C2H6O	46.1	522.2	104.023	1.17009	dimer
25	2-Butanone	C78933	C4H8O	72.1	586.5	132.112	1.06086	monomer
26	2-Butanone	C78933	C4H8O	72.1	587.8	132.685	1.24907	dimer
27	Hexanal	C66251	C6H12O	100.2	789.6	258.95	1.25527	monomer
28	Hexanal	C66251	C6H12O	100.2	788.8	258.092	1.56034	dimer
29	2-Methylpropanol	C78831	C4H10O	74.1	624.8	148.857	1.16607	monomer
30	2-Methylpropanol	C78831	C4H10O	74.1	624.8	148.857	1.36238	dimer
31	(E)-2-Pentenal	C1576870	C5H8O	84.1	744.1	220.997	1.10807	monomer
32	(E)-2-Pentenal	C1576870	C5H8O	84.1	746.2	222.68	1.35897	dimer
33	1-Butanol	C71363	C4H10O	74.1	664.2	166.036	1.179	
34	3-Methylbutanol	C123513	C5H12O	88.1	736.6	215.045	1.49618	dimer
35	3-Methylbutanol	C123513	C5H12O	88.1	731.4	210.897	1.79254	polymer
36	Isopentyl Acetate	C123922	C7H14O2	130.2	869.9	347.375	1.30617	monomer
37	Isopentyl Acetate	C123922	C7H14O2	130.2	869.5	346.96	1.75	dimer
38	Ethyl Propanoate	C105373	C5H10O2	102.1	701.9	187.341	1.45434	
39	Butanal	C123728	C4H8O	72.1	592	134.505	1.29064	
40	Dimethyl Trisulfide	C3658808	C2H6S3	126.3	954.7	482.904	1.31729	
41	3-(Methylthio) Propanol	C505102	C4H10OS	106.2	984.9	536.605	1.464	
42	Octanal	C124130	C8H16O	128.2	1005.1	574.034	1.40534	
43	n-Nonanal	C124196	C9H18O	142.2	1106.2	770.91	1.47695	
44	Ethyl Pentanoate	C539822	C7H14O2	130.2	896.9	380.317	1.27255	
45 *	2-Methyl-3-Heptanone	C13019200	C8H16O	128.2	930.7	440.309	1.27858	monomer
46 *	2-Methyl-3-Heptanone	C13019200	C8H16O	128.2	930.7	440.309	1.69262	dimer

^a^ Represents the molecular mass; ^b^ Represents the retention index calculated using n-ketones C4–C9 as external standard on MXT-5 column; ^c^ Represents the retention time in the capillary GC column; ^d^ Represents the drift time in the drift tube; * Internal standard substance; ^#^ Represents the Chemical Abstracts Service.

**Table 2:** Volatile compounds in four stages of Zhenba bacon by GC–MS.

Compound	Number	CAS	RI	Fresh	YZ	XZ	CP
**Ketones**							
3-Methyl-1,2-Cyclopentanedione	M1	765-70-8	1631.1	——	——	0.449 ± 0.11	0.329 ± 0.14
3-Methyl-2-Cyclopenten-1-One	M2	2758-18-1	——	——	——	0.275 ± 0.21	——
2,3-Octadione	M3	585-25-1	1334.7	——	0.287 ± 0.23	——	——
2,3-Dimethyl-2-Cyclopentene-1-One	M4	1121-05-7	1538.1	——	0.078 ± 0.02	——	——
**Phenols**							
2,5-Dimethylphenol	M5	576-26-1	2099.1	——	——	——	0.209 ± 0.09
2-Methylphenol	M6	95-48-7	2022.7	——	——	0.487 ± 0.13	0.362 ± 0.17
3-Methylphenol	M7	108-39-4	——	——	——	0.599 ± 0.26	0.696 ± 0.22
4-Methyl Guaiacol	M8	93-51-6	1694.0	——	——	0.522 ± 0.03	0.415 ± 0.11
4-Ethylguaiacol	M9	2785-89-9	2059.9	——	——	0.279 ± 0.12	0.221 ± 0.03
Guaiacol	M10	90-5-1	1647.7	——	——	0.735 ± 0.32	0.567 ± 0.27
Phenol	M11	108-95-2	2028.9	——	——	1.136 ± 0.46	0.869 ± 0.29
**Alcohols**							
Ethanol	M12	64-17-5	939.6	——	16.017 ± 3.74	3.982 ± 1.01	7.084 ± 2.32
Phenethyl Alcohol	M13	60-12-8	1673.0	——	0.135 ± 0.08	——	——
3-Octenol	M14	3391-86-4	1627.4	——	0.365 ± 0.43	0.129 ± 0.03	0.214 ± 0.01
Furfuryl Alcohol	M15	98-00-0	1588.3	——	——	0.320 ± 0.09	0.241 ± 0.01
2-Methyl-1-Propanol	M16	78-83-1	1098.2	——	0.489 ± 0.23	——	0.103 ± 0.01
3-Methyl-1-Butanol	M17	123-51-3	1214.0	——	5.757 ± 1.51	0.83 ± 0.03	1.643 ± 0.16
1-Hexanol	M18	111-27-3	1360.1	——	0.146 ± 0.05	——	——
1-Pentanol	M19	71-41-0	1256.3	——	0.332 ± 0.07	0.083 ± 0.01	0.063 ± 0.03
**Esters**							
Ethyl Acetate	M20	141-78-6	888.0	——	——	0.193 ± 0.01	——
Ethyl Octanoate	M21	106-32-1	1606.4	——	——	——	0.091 ± 0.02
Ethyl Valerate	M22	539-82-2	1129.4	——	0.187 ± 0.03	——	——
Ethyl Caproate	M23	123-66-0	1241.8	——	1.131 ± 0.29	0.258 ± 0.11	0.311 ± 0.01
Methyl Hexanoate	M24	106-70-7	1194.4	——	0.718 ± 0.37	0.790 ± 0.35	0.614 ± 0.19
Ethyl Caprate	M25	110-38-3	1574.6	——	——	0.191 ± 0.01	——
Ethyl Butyrate	M26	105-54-4	1041.8	——	0.342 ± 0.02	0.102 ± 0.02	0.168 ± 0.03
Methyl Butyrate	M27	623-42-7	990.1	——	——	0.460 ± 0.27	0.375 ± 0.11
Ethyl Lactate	M28	97-64-3	1356.9	——	0.104 ± 0.03	——	0.082 ± 0.03
**Aldehydes**							
Pentanal	M29	110-62-3	986.6	——	0.112 ± 0.01	——	——
Hexanal	M30	66-25-1	1091.0	0.203 ± 0.03	3.496 ± 0.85	0.834 ± 0.17	0.892 ± 0.26
1-Nonanal	M31	124-19-6	1419.2	——	0.323 ± 0.03	0.203 ± 0.03	0.153 ± 0.01
Furfural	M32	98-1-1	1680.2	——	——	0.762 ± 0.21	0.562 ± 0.01
5-Methyl Furfural	M33	620-02-0	1552.3	——	0.760±0.01	0.244 ± 0.03	0.173 ± 0.01
Acetal	M34	105-57-7	893.2	——	0.458±0.03	——	——
**Others**							
1,4-Dimethoxybenzene	M35	150-78-7	1767.6	——	0.158 ± 0.01	0.114 ± 0.02	0.185 ± 0.03
Naphthalene	M36	91-20-3	1784.9	——	——	0.105 ± 0.01	——
2-Acetylfuran	M37	1192-62-7	1516.7	——	0.326 ± 0.02	0.114 ± 0.02	——
Acetic Acid Glacial	M38	64-19-7	1640.4	——	0.742 ± 0.13	0.621 ± 0.24	0.448 ± 0.09
Pyridine	M39	110-86-1	1200.5	——	——	0.389 ± 0.01	——
Butyric Acid	M40	107-92-6	1570.6	——	——	0.104 ± 0.01	——

RI: Retention indexes; Represents the Chemical Abstracts Service; Fresh: raw sample; YZ: cured sample; XZ: smoking sample; CP: finished sample.
